# Numerical and Experimental Research on the Brushing Aluminium Alloy Mechanism Using an Abrasive Filament Brush

**DOI:** 10.3390/ma14216647

**Published:** 2021-11-04

**Authors:** Xiuhua Yuan, Chong Wang, Qun Sun, Ling Zhao

**Affiliations:** School of Mechanical and Automotive Engineering, Liaocheng University, Liaocheng 252000, China; yuanxiuhua@lcu.edu.cn (X.Y.); wangchong@lcu.edu.cn (C.W.); zhaoling@lcu.edu.cn (L.Z.)

**Keywords:** abrasive filament brush, brush grinding, material removal, brush force, finite element approach

## Abstract

Abrasive filament brushes have been widely used in surface processes for a wide range of applications, including blending, edge-radiusing, and polishing. However, the associated brush mechanics of material removal is still not clear. In order to analyze the brush grinding of aluminium alloy, this paper constructed a kinematic model of a single filament, simulated the scratch process of a single abrasive grain, and investigated the brush force and material removal based on the finite element approach. The simulated result shows that the brush grinding can be changed from elastic–plastic deformation to chip formation when increasing the brush speed to 1000 r/min. The normal and tangential forces increase linearly and quadratically with the increase in the rotation speed (500–5000 r/min), respectively, and increase linearly with the increase in the penetration depth (0.1–1 mm), which is consistent with the experiment results. In addition, the amount of material removal initially increases with the increase in penetration depth, and then decreases. This paper provides a new approach to understanding the process of material removal and is helpful for the selection of reasonable brush parameters in the intelligent grinding control application.

## 1. Introduction

**Figure 1:**
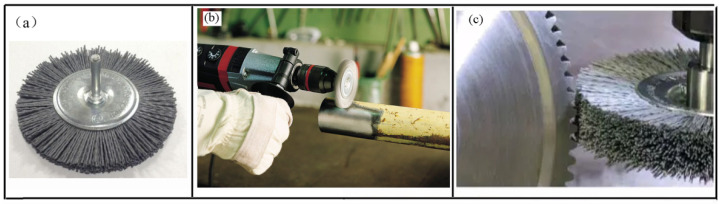
Abrasive filament brush (**a**); brush derusting (**b**); and grinding gear (**c**).

Brush grinding is a surface treatment using rotating tools [1,2]. Abrasive filament brushes are made of abrasive particle-filled polymer, which can grind materials with high hardness. As shown in Figure 1, the abrasive filament brush can effectively carry out surface treatment, such as burr removal [3,4], derusting, polishing [5], and edge-radiusing [6]. Currently, the abrasive filament brush is widely used in hand-held grinding machines without comprehensive control techniques, and the intelligent abrasive robot with brush is still rare in the market because of the difficulty in controlling the end effector [7]. The use of a filament brush in the intelligent automation environment requires a clear understanding of brush performance, such as brush force, the material removal rate, and so on [8].

In order to explore the grinding mechanism of abrasive filament brushes, many researchers have carried out brush-grinding experiments on different materials. Overholser et al. examined the surface morphology of a 6061-T6 workpiece after brush grinding and found that the primary material removal was abrasive cutting and chip formation [9]. Raymond et al. produced a functional surface-of-sliding guideway and found that brush grinding can reduce both the height and volume of asperities on the milled surface [10,11]. Mathai et al. investigated the brush deburring of Nitinol foil and found that the burr removal can be divided into two stages: fatigue fracture and crack formation [12,13]. Novotný et al. presented the novel process of frosted glass using an abrasive filament brush and provided a good quality surface [14]. The above foregoing sources mainly focused on the surface morphology changes after brush grinding and do not clarify the influence of the process parameters on the brush force and material removal rate.

When removing rust with a steel wire brush, our group constructed a brush force model assuming that the tip of the wire moved along the rigid surface; that is, the ratio of normal force to the tangential force was constant [15]. However, in the process of brush grinding, not only elastic–plastic deformation occurs, but chips are also produced. On the other hand, the machining marks of the aluminium alloy workpiece, resulting from ball-milling, can be removed by an abrasive filament brush. In order to explore the associated brush mechanics of material removal, this paper constructed the kinematic model of a single filament, carried out the brush grinding of aluminium alloy based on the finite element approach, and analyzed the effect of the process parameters on the brush force and material removal rate.

## 2. Analysis of the Impact Phase

**Figure 2:**
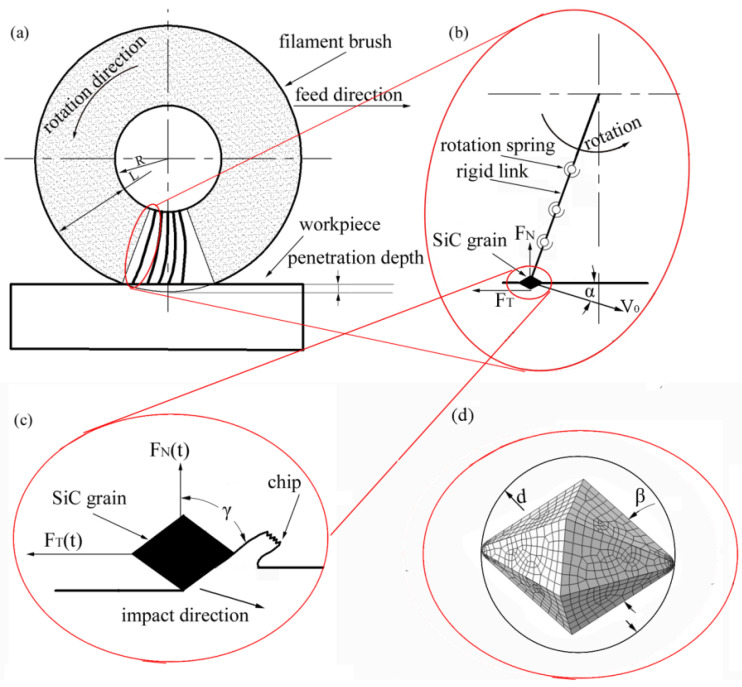
Brush grinding: (**a**) filament brush; (**b**) single abrasive filament; (**c**) impact phase of grain; and (**d**) the geometry of abrasive grain after meshing.

As shown in Figure 2a, brush grinding is a type of multipoint cutting, where a large number of abrasive grains participate in the cutting process at the same time. The filaments are placed radially from the hub center and are limited to the outside radius of the brush. Under ideal conditions, there is no interaction between different abrasive filaments during brush grinding. Without losing the generality, the study of the single filament grinding process can effectively investigate the complex brush process, so as to reveal the material removal mechanism. As shown in Figure 2b, the filament was divided into rigid links and joints, with rotating springs and rotating dampers. Because the contact lengths and times between the abrasive grains and the workpiece were very short in the impact process, it was assumed that the elastic coefficient and damping coefficient at the joint were zero. The detailed collision process at the end of the filament is shown in Figure 2c. The SiC grain was generally assumed to be a polyhedral model because it was made of crystalline ceramic with ionic or covalent atomic bonds. On the basis of the above consideration, this paper hypothesized that the SiC grain in the filament was dodecahedron (Figure 2d), which consisted of an orthohexagon and eight vertexes. In addition, the SiC grain had the cone angle, β, and diameter, *d*. The SiC grains played a key collision role at the moment of contact, as their hardness was higher than that of the filaments. The kinetic energy of the SiC grain acted on the local area (impact) of the workpiece surface, resulting in the generation of the microcrater and chips. As shown in Figure 2b, the impact velocity and impact energy of the SiC grain at the moment of contact with the workpiece can be calculated as follows:

*V*_0_ = ((*R* + *L*) × 2π*n*/1000 + *f*)/60 (1)

*E* = 1/2 *m V*_0_^2^ = 1/2 *m* (((*R + L*) × 2π*n*/1000 + *f*)/60)^2^ (2)

where *m* is the weight of the SiC grain (kg); *R* is the hub radius (mm); *L* is the filament length (mm); *n* is the the rotation speed (r/min); and *f* is the feed rate (m/min). As shown in Figure 2b, the impact angle, *α*, of the SiC grain at the moment of contact can be calculated as follows:

*α* = acrcsin ((*R* + *L* − Δ)/(*R + L*)) (3)

where *R* is the hub radius (mm); *L* is filament length (mm); and Δ is the penetration depth (mm). The normal and tangential components of the impact velocity at the moment of impact are shown as follows:

*V_N_* = *V*_0_sin*α* = (((*R* + *L*) × 2π*n*/1000 + *f*)/60) × ((*R* + *L* − Δ)/(*R* + *L*)) (4)

*V_T_* = *V*_0_cos*α* = (((*R* + *L*) × 2π*n*/1000 + *f*)/60) × cos (arcsin ((*R* + *L* − Δ)/(*R* + *L*))) (5)

where *R* is the hub radius (mm); *L* is the filament length (mm); and Δ is the penetration depth (mm). During the impact process, the rake angle, *γ,* of the SiC grain (shown in Figure 2c) was influenced by the shape of the SiC grain and the cutting direction. During brush grinding, the brush force of a single grain was also composed of the normal component force, *F_N_*, and the tangential component force, *F_T_*. The normal component force, *F_N_*, and the tangential component force, *F_T_*, of a single particle can be calculated as follows:

(6) $$F_{N}=\frac{n}{60}\int_{0}^{\frac{60}{n}}F_{N}\left(t\right)dt$$

(7) $$F_{T}=\frac{n}{60}\int_{0}^{\frac{60}{n}}F_{T}\left(t\right)dt$$

where *n* is the rotation speed of a single grain (r/min); *t* is the time (s); *F_N_*(*t*) is the normal component force (N) at the time of *t*; and *F_T_*(*t*) is the tangential component force (N) at the time of *t*. The impact process of the abrasive grain is a fast dynamic process, so it is difficult to accurately calculate the brush force by analytical methods. In this paper, the impact process of the abrasive grain was simulated using finite element analysis, and the values of the instant force, *F_N_*(*t*)*,* and *F_T_*(*t*) at different times can be obtained from the simulation result of the impact process. The integral values of $\int_{0}^{\frac{60}{n}}F_{N}\left(t\right)dt$ and $\int_{0}^{\frac{60}{n}}F_{T}\left(t\right)dt$ were calculated by the numerical integration method.

From the above analysis, it can be seen that the velocity of the SiC grain at the moment of contact was composed of normal and tangential component velocities, which were mainly influenced by the rotation speed and the penetration depth. During the impact process, the brush force was also composed of the normal force and the tangential component force, which were influenced by the particle energy and the impact angle. That is, the normal force and the tangential component force were mainly influenced by the rotation speed and the penetration depth. However, the impact of the abrasive grain is a fast dynamic process, so it is difficult to accurately calculate the brush force and material removal rate by analytical methods. The finite element simulation can be employed to solve implicit problems and is widely used in the fields of structural strength, fluid analysis, and metal cutting. Therefore, this paper applied the Abaqus (a type of finite element analysis product, [16,17,18]) to simulate the impact process of the abrasive grain, and to calculate the brush force and material removal rate.

## 3. Experimental Setup

**Figure 3:**
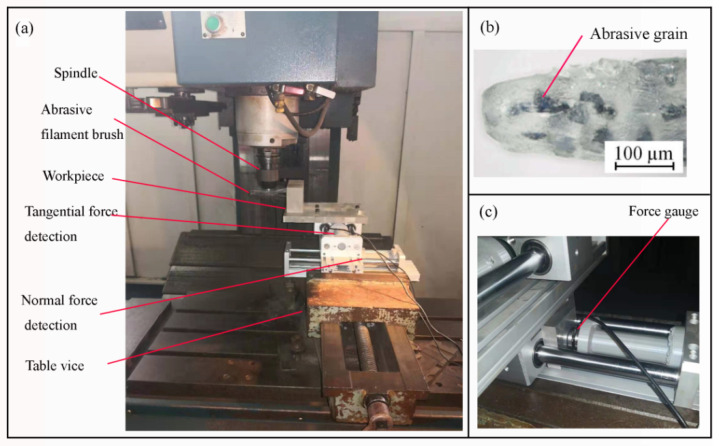
Experimental setup (**a**); abrasive filament; (**b**) and force measure (**c**).

The workpiece material was 7075 aluminium alloy, which is widely used in the thin-walled parts of the aviation industry. Before brush grinding, all specimens were milled with a ball-end cutter (diameter 20mm). As shown in Figure 3, the brush grinding treatment was performed with a vertical machine center, an abrasive filament brush and force-measuring component. The abrasive filament brush has the hub radius, R = 50 mm, the filament length, L = 50 mm, the filament diameter, d = 0.4 mm, an abrasive grain size of 400 mesh, a particle diameter, d = 40 µm, and the particle weight, m = 2 × 10^−10^ kg. As shown in Figure 3b [19], abrasive grains were randomly distributed within the filament. The force-measuring component was composed of a load sensor (Figure 3c) and a signal amplifier, which can read the normal and tangential forces with a measuring accuracy of 0.01 N. The surface topography was measured by Wyko NT9800 optical profiler (Veeco Instruments Inc., Oak Ridge, TN, USA).

**Figure 4:**
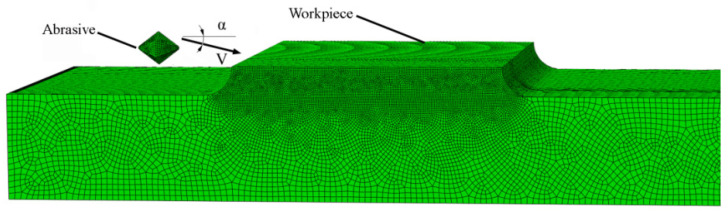
Abrasive brush grinding model.

The finite element simulation can be employed to solve implicit problems and is widely applied in the fields of structural strength, fluid analysis, and metal cutting [16,17]. Therefore, this paper applied the Abaqus software (a type of finite element analysis product, [18]) to simulate the brush grinding process. The geometrical model of the workpiece and the abrasive grain are shown in Figure 4. The parameters of the abrasive grain model were diameter, d = 40 μm; the cone angle, β = 80°; the rake angle, γ = 50°; and the grain weight, m = 2 × 10^−10^ kg. The abrasive grain was assumed to be a rigid body owing to its much higher hardness than that of the aluminium alloy. The workpiece was made of 7075 aluminium alloy, whose density, Poisson’s ratio, and Young’s modulus were 2.8 g/cm^3^, 0.3, and 71 GPa, respectively. The Johnson–Cook constitutive equation (detailed parameters: A = 470 MPa, B = 331 MPa, n = 0.34, C = 0.012, and m = 0.8) was selected as its material constitutive model. The Johnson–Cook damage equation (detailed parameters: d_1_ = −0.09, d_2_ = 0.25, d_3_ = −0.5, d_4_ = 0.014, d_5_ = 3.87, θ_melt_ = 600 °C, and θ_transition_ = 20 °C) was selected as its damage model. In damage evolution, the displacement at failure is 0.08. In the mesh module, the refined element size of 2 μm was chosen for the contact zone, and an eight-node hexahedron element was applied to the meshing. In the interaction module, the surface-to-surface contact was selected as the interaction type, and the penalty function was chosen to be the interaction property. In the boundary condition, the workpiece was constrained on the bottom side, and the abrasive grain was constrained at four degrees of freedom, except at the normal and tangential directions. We examined the impact performance of the 7075 aluminium alloy, and the brush parameters in the simulation are given in Table 1. The normal and tangential component velocities at the moment when the abrasive grain just contacted the workpiece can be calculated by Equations (1)–(5), and these were inputted into the Abaqus software as the initial state of the abrasive grain for the simulation of the impact process. The initial positions of the abrasive grain and the workpiece were invariant under different process parameters.

## 4. Results

### 4.1. Surface Topography after Brush Grinding Experiment

**Figure 5:**
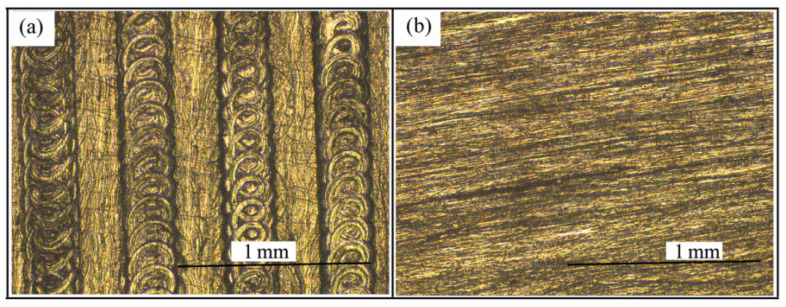
Surface texture before (**a**); and after (**b**) brush grinding experiment.

**Figure 6:**
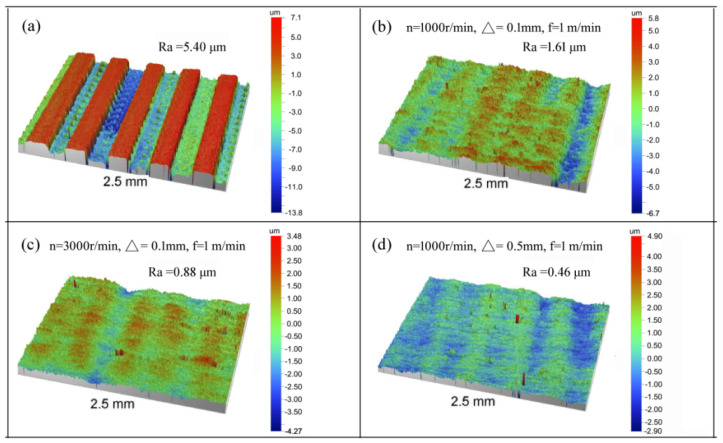
The experiment morphology (**a**) before brush grinding, and the experiment morphology (**b**–**d**) after brush grinding, at different process parameters.

In order to understand the brush grinding more comprehensively, brush grinding experiments under different process conditions were carried out. The surface topographies of the aluminium alloy before and after brush the grinding experiment are shown in Figure 5 and Figure 6. As shown in Figure 5a and Figure 6a, the surface topography before brush grinding clearly illustrates that the translation of the workpiece and the rotational motion of the ball-end cutter caused the peak and valley. As shown in Figure 6b, the surface roughness after brush grinding changed from Ra = 5.4 μm to Ra = 1.61 μm, and the peak was reduced because of the impact effect of the abrasive grain. When the brush speed and penetration depth were further increased, the surface roughness was reduced to Ra = 0.88 μm and Ra = 0.46 μm, respectively, which is shown in Figure 6c,d. From the above analysis, it can be seen that the filament brush can effectively remove the majority of the surface peaks, and the surface was smoother than the milled surface.

### 4.2. Aluminium Alloy Removal Mode

**Figure 7:**
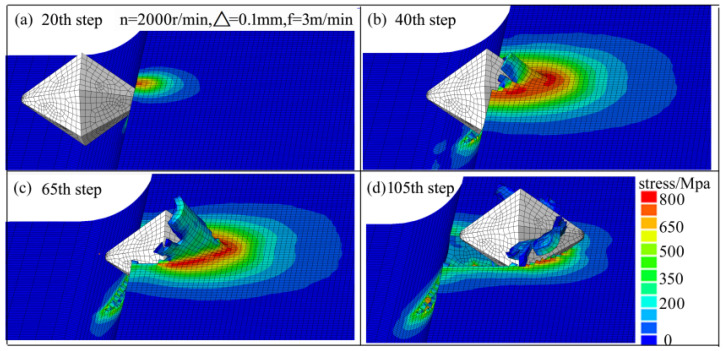
Stress field and chip formation in abrasive impact process: (**a**) 20th time step; (**b**) 40th time step; (**c**) 65th time step; (**d**) 105th time step.

The impact process of the abrasive grain is shown in Figure 7. When the simulation ran to the 20th time step, the aluminum alloy suffered from the impact of the grain, and the stress in the contact area was higher than that in other areas. Moreover, the maximum extrusion stress of the aluminum alloy increased sharply to 700 MPa, and plastic deformation appeared. As the abrasive grain invaded the aluminium alloy, the extrusion pressure between them also increased continuously. When the simulation ran to the 40th time step, the scratches and plastic deformation increased, and the chips appeared on the aluminium alloy. When the simulation ran to the 65th time step, the plastic deformation and chips were further intensified. The whole impact process was completed at the 105th time step, when the particle detached from the aluminum alloy. Therefore, the impact process of the abrasive grain was composed of plastic deformation, scratch, and chip formation.

**Figure 8:**
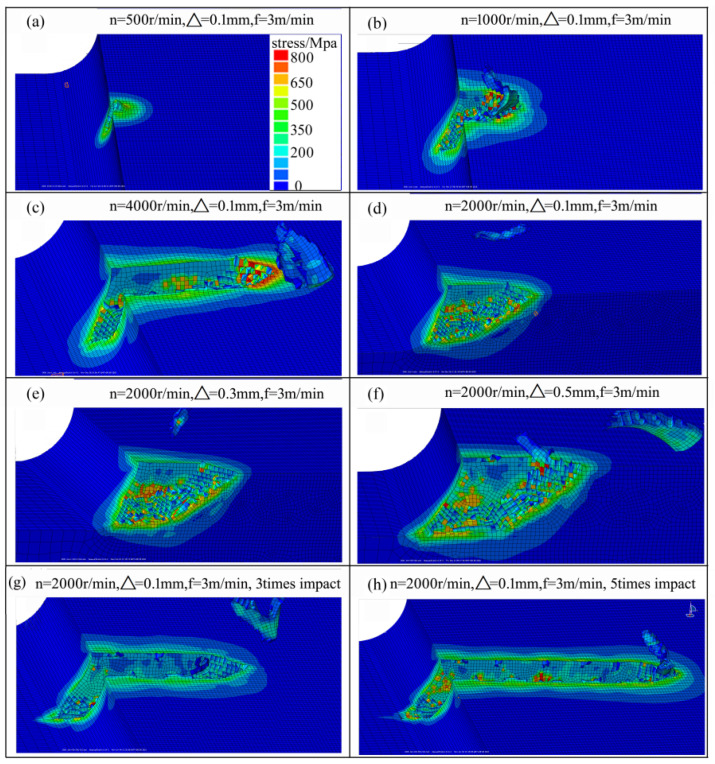
The material removal after the impact of SiC particle: (**a**–**c**) through increasing the revolution speed; (**d**–**f**) through increasing the penetration depth; (**g**,**h**) through increasing the number of impact processes.

Figure 8 shows the change in the behavior of material removal under different process parameters. As shown in Figure 8a–c, when the brush speed was 500 r/min, the tensile stress of the aluminium alloy did not reach to the yield stress because of the lower impact energy of the abrasive grain; thus, there was only elastic–plastic deformation and no obvious chip generation. When the brush speed increased from 500 r/min to 4000 r/min, the aluminium alloy surface was torn, and the amount of chips removed from the aluminium alloy also increased. As shown in Figure 8d–f, when the penetration depth increased from 0.1 mm to 0.5 mm, the impact length in the aluminium alloy surface increased, and the amount of materials removed from the aluminium alloy also increased. As shown in Figure 8g,h, when the number of impacts increased, the amount of material removed from the aluminium alloy also increased. From the above analysis, it can be seen that the process parameters can affect the brush force and the amount of material removal.

### 4.3. Brush Force

**Figure 9:**
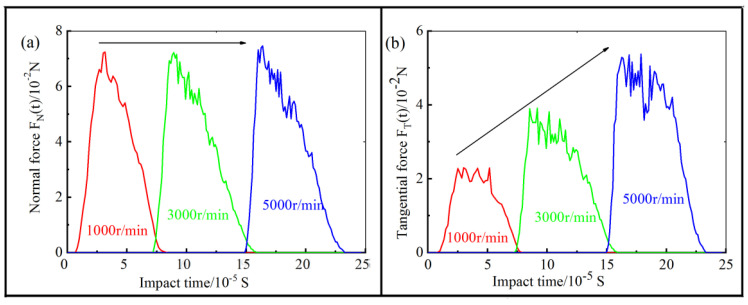
The simulation results of single abrasive grain during impact process: (**a**) the normal force *F_N_*(*t*); and (**b**) tangential force *F_T_*(*t*).

**Figure 10:**
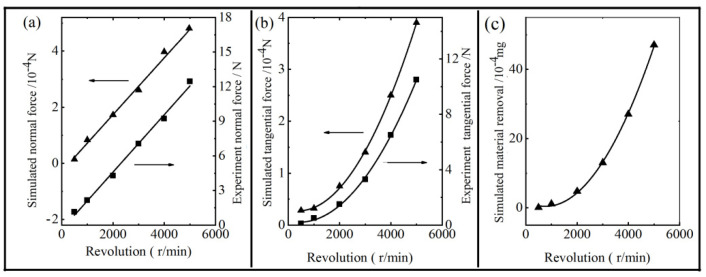
The effect of revolution speed on the normal force (**a**); tangential force (**b**); and the mass of material removal (**c**).

In order to establish a mathematical end-effector model that can predict brush force for intelligent control, an assessment of the influence of the process parameters on brush force was carried out. For the penetration depth of 0.1 mm, the change of force with time during the impact process is shown in Figure 9a,b. From Figure 9a, it can be seen that the instantaneous normal force, *F_N_*(*t*), of a single abrasive grain hardly changed with the increase in the brush speed, which is because the normal force was mainly influenced by the penetration depth and the rake angle of the abrasive grain. As shown in Figure 9b, the instantaneous tangential force, *F_T_*(*t*), of the abrasive grain during the impact process increased linearly with the increase in brush speed. Figure 10 shows the influence of the revolution speed on the brush force at a constant penetration depth of 0.1 mm. The simulated tangential force and normal force of the abrasive grain can be calculated by Equations (6) and (7), and the experimental force was tested by the force-measuring component. It can be seen that the simulated normal force increases significantly with the increase in the brush speed. In addition, the relationship between the normal force and the brush speed was linear polynomial function, which is because the influenced factors in Equation (6) were the brush speed, n, and the integral of *F_N_*(*t*), and the integral of *F_N_*(*t*) were constant. The relationship between the tangential force and the brush speed was a quadratic polynomial function, which is because the influenced factors in Equation (7), the brush speed, n, the integral of *F_T_*(*t*), and the integral of *F_T_*(*t*), were also linearly increased with the increase in the brush speed. The brush force from the experiment had the same change trend as that from the simulation, which was similar to that reported in another article [13]. Moreover, it was about 2 × 10^4^ times larger than the simulated value, which is because the thousands of abrasive grains bonding to the bundles of the nylon bristles simultaneously impacted on the workpiece surface. Therefore, the brush forces from the experiment were directly proportional to those from the simulation. As shown in Figure 10c, the mass of material removal increased quadratically with the increase in brush speed. When the brush speed of the abrasive grain reached up to 5000 r/min, the mass of material removal grew up to the maximum. Therefore, the mass of material removal increased with the increase in the brush speed of the grain, but could not increase without limit, because the strength of the filament was certain.

**Figure 11:**
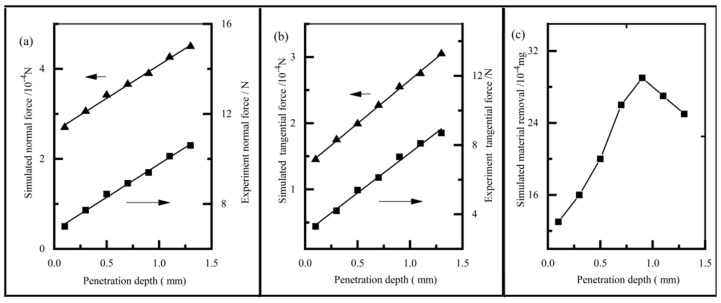
The effect of penetration depth on the normal force (**a**); tangential force (**b**); and material removal (**c**).

Figure 11 shows the influence of the penetration depth on the brush force at a constant brush speed of 3000 r/min. The simulated tangential force and the normal force of the abrasive grain can be calculated by Equations (6) and (7), and the experiment force was tested by the force-measuring component. It can be seen that both the normal and tangential forces in the simulation increased significantly with the increase in the penetration depth. Furthermore, both the tangential and normal forces exhibited linear polynomial function with the penetration depth, mainly due to the increase in the impact angle, α, of the abrasive grain, which was similar to that reported in another study [19]. Figure 11c shows the relationship between the penetration depth and the mass of material removal. It can be seen that the mass of material removal firstly grew up, and then grew down with the increase in the penetration depth. When the impact angle increased, the abrasive grain better overcame the vertical component force because of its lower rake angle, resulting in more chip formation. Nevertheless, when the impact angle was larger than 10°, the abrasive grain could better penetrate into the matrix, leading to larger plastic deformation and less chip formation.

## 5. Conclusions

In order to analyze the brush grinding mechanism of material removal, a kinematics model of a single filament was established and the scratch process of the abrasive grain was simulated. Moreover, the brush force and material removal under different brush speeds and penetration depths were studied, based on the finite element method. According to the experimental and simulation results, we drew the following conclusions related to the brush behavior:

(1) During the brush grinding of aluminium alloy, only plastic deformation and scratches could be observed when the brush speed was less than 500 r/min. When the rotation speed was greater than 1000 r/min, the chip deformation appeared.

(2) The normal and tangential forces increased linearly and quadratically with the increase in rotation speed, respectively. In addition, the material removal increased quadratically with the increase in rotation speed.

(3) The tangential force and the normal force increased linearly with the increase in the penetration depth. In addition, the material removal rate increased with the increase in the penetration depth, and then decreased. The variation trend of the simulated brush force agreed well with that of the experiment brush force, which can provide guidance for brush grinding for intelligent grinding control application.

The purpose of this paper was to develop an intelligent mobile robot system for brushing and grinding workpieces without manual control. Through the finite element analysis of the impact process, the laws between the brush force, the material removal rate, and the process parameters were revealed. However, because of the wear of the filament, the brush grinding robot is difficult to control. Therefore, the next step is to develop an intelligent prediction module of the surface quality that comprehensively considers the process parameters and the brush force using an artificial neural network.

## Figures and Tables

**Table 1:** The process parameters of brush grinding.

No	Revolution Speed (n r/min)	Penetration Depth (Δ mm)	Feed Rate (f m/min)
1	500	0.1	3
2	1000	0.1	3
3	2000	0.1	3
4	3000	0.1	3
5	4000	0.1	3
6	5000	0.1	3
7	3000	0.3	3
8	3000	0.5	3
9	3000	0.7	3
10	3000	0.9	3
11	3000	1.1	3
12	3000	1.3	3
13	1000	0.1	1
14	3000	0.1	1
15	3000	0.5	1

## Data Availability

The authors confirm that the data supporting the findings of this study are available within the article.
